# Effects of Dietary Fish Meal Replacement with Alternative Protein Ingredients and Their Combinations on Growth Performance, Feed Utilization, Fillet Composition, and Biochemical Parameters of Red Seabream (*Pagrus major*)

**DOI:** 10.1155/2023/8883739

**Published:** 2023-07-13

**Authors:** Buddhi E. Gunathilaka, Seong-Mok Jeong, Min-Uk Cho, Kang-Woong Kim, Sang-Woo Hur, Seunghan Lee, Sang-Guan You, Sang-Min Lee

**Affiliations:** ^1^Department of Aquatic Life Medicine, Gangneung-Wonju National University, Gangneung 25457, Republic of Korea; ^2^Aquafeed Research Center, National Institute of Fisheries Science, Pohang 37517, Republic of Korea; ^3^Department of Marine Food Science and Technology, Gangneung-Wonju National University, Gangneung 25457, Republic of Korea

## Abstract

The experiment was conducted to evaluate alternative protein ingredients in a low-fish meal (FM) diet for red seabream (*Pagrus major*). Twelve experimental diets were formulated. Control diet (CON) was designed to contain 60% FM. Other experimental diets were formulated by replacing 50% of FM from the CON with soy protein concentrate (SPC), corn gluten (CG), meat meal (MM), and/or chicken byproduct meal (CBM). Four diets were designed including one of SPC, CG, MM, or CBM as FM replacer and designated as SPC, CG, MM, and CBM. Six other diets were formulated by adding two ingredients as SPC and CG, SPC and MM, SPC and CBM, CG and MM, CG and CBM, or MM and CBM, and designated as SCG, SMM, SCM, CMM, CCM, and MCM, respectively. The 12th diet (MIX) was formulated by including SPC, CGM, MM, and CBM. Triplicate fish groups (50.2 ± 0.1 g) were hand-fed for 12 weeks. Weight gain (WG) of fish was significantly improved by MM and MCM diets compared to CG, SCG, CMM, and CCM diets. WG of CON, SPC, CM, SMM, SCM, and MIX groups were comparable with MM and MCM groups. The lowest WG was observed in CG and CMM groups. Feed efficiency (FE) was significantly higher in MM group compared to SPC, CG, SGC, and CMC groups. FE of MCM group was significantly higher than CG and SCG groups. Fillet linolenic acid (C18:2*n–*6) level in CG group was significantly higher than CON, MM, CM, SCM, CCM, and MCM groups. Serum lysozyme activity was significantly higher in MCM and MIX groups. Therefore, a high level of dietary CG reduces the growth performance and feed utilization of red seabream. A mixture of MM and CBM seems to be more efficient in replacing FM from red seabream diet.

## 1. Introduction

Alternative protein ingredients have been evaluated in fish feed during the last few decades because of limited supply, high demand, and increasing price of fish meal (FM). Different types of alternative protein sources such as plant-based byproducts, terrestrial animal byproducts, insect meals and single-cell proteins are currently used in the aquafeed sector [1–4].

Soy protein concentrate (SPC) has been evaluated as an alternative protein source in red seabream (*Pagrus major*) diets through a number of research works. SPC could be used to replace some extent of FM from red seabream diets [5]. Thereafter, it was required to add functional ingredients, limiting amino acids, and/or developed protein sources to maintain performance of red seabream fed diets containing a high level of SPC [6–8]. However, growth performance of red seabream was retarded due to reduced feed intake and lipid accumulation when SPC was used as the sole dietary protein source [9].

Corn gluten (CG) is also incorporated in red seabream diets when formulating low-FM diets [8, 10]. However, there is a lack of information about CG as FM replacer in red seabream diet. Several studies reported that CG can be effectively used together with other protein sources for FM replacement in red seabream diet [11, 12]. Aoki et al. [11] concluded that red seabream diet should not contain CG over 10%. However, CG was successfully used to replace 20%–40% of FM protein from black seabream (*Acanthopagrus schlegelii*) diet and 60% of FM protein from gilthead seabream (*Sparus aurata*) diet [13, 14].

Meat meal (MM) is produced using byproducts discarded from meat processing plants and slaughterhouses. It contains a high level of protein as it is produced without bones [15]. Information about the usage of MM in fish diet is limited because meat byproducts were processed with bones to prepare meat and bone meals in many cases. According to available information, Aoki et al. [11] observed that a mixture of MM, CG, and soybean meal could replace 46%–62% of FM in red seabream diets. In juvenile grouper (*Epinephelus coioides*) diet, 80% of FM was successfully replaced with a mixture of MM and blood meal [16]. Williams et al. [17] evaluated two different MM products having 52% and 60% crude protein and observed improved growth performance and feed utilization in barramundi (*Lates calcarifer*). MM successfully replaced approximately 70% of FM from olive flounder (*Paralichthys olivaceus*) diet [18]. Nutrient digestibility of MM in olive founder was also reported as higher than several marine-based feed ingredients [15].

Chicken byproduct meal (CBM) is also produced from processing waste such as necks, feet, and intestines. An early study reported that CBM could replace dietary FM up to 100% in yearlings and up to 70% in juvenile stage of red seabream without adverse effects [19]. However, CBM was not examined further in red seabream diet as a FM replacer although information is available on other species in seabream family (Sparidae). CBM replaced up to 83% of FM in gilthead seabream diet without sacrificing growth and feed utilization [20]. Dietary CBM did not compromise welfare and fillet quality of gilthead seabream [21]. Randazzo et al. [22] reported that CBM can be supplemented in gilthead seabream diet as an alternative to plant protein because of improved gut status reducing the inflammatory markers and improving lipid absorption. Expression of genes involved in protein metabolism was upregulated in back seabream fed a diet prepared by replacing 30% of FM with CBM [23]. Moreover, recent studies on other fish species reported positive roles of CBM such as improving nutrient digestibility in black sea bass (*Centropristis striata*) diet [24], enhancing gut and liver health of rainbow trout (*Oncorhynchus mykiss*) fed non-FM diets [25], and maintaining immune status of olive flounder fed 50% of FM replaced diets [26].

Red seabream is a carnivorous fish species cultured in the Eastern Asia region. Average red seabream production in South Korea was estimated as 5,400 tons per year [27]. The limitations of red seabream aquaculture are known as high-feed cost, low-feed efficiency under suboptimal temperatures and disease outbreaks. They require approximately 45%–50% crude protein in diets including marine originated protein for better production [28, 29]. Alternative protein sources were evaluated in red seabream diet in several studies to reduce feed costs under both optimal and suboptimal temperatures [28, 30]. It is well-documented that dietary FM replacement with mixtures of protein sources was efficient for carnivorous fish species including red seabream [31, 32]. Therefore, we designed the present study supplementing SPC, CG, MM, and/or CBM in a low-FM diet to find the efficient ingredients or their combination on growth performance, feed utilization, fillet composition, and biochemical parameters of red seabream.

## 2. Materials and Methods

### 2.1. Experimental Diets

Twelve experimental diets were formulated to be isonitrogenous (45% crude protein) and isocaloric (18 kJ g^−1^) as shown in Table 1. The control diet (CON) was designed to contain 30% tuna byproduct meal, 15% sardine meal, and 15% pollock meal. Eleven other experimental diets were formulated to contain 30% FM by replacing 50% of FM from the CON including 15% tuna byproduct meal, 7.5% sardine meal, 7.5% pollock meal, and alternative protein sources to provide reduced protein level after FM replacement. Four diets were designed to contain SPC, CG, MM, or CBM at 30.2%, 30.8%, 24.1%, or 31.2% inclusion levels. Six diets were designed as SCG, SMM, SCM, CMM, CCM, and MCM by including two alternative protein ingredients. SCG diet contained 15.1% SPC and 15.4% CGM. SMM diet contained 15.1% SPC and 12.1% MM. SCM diet contained 15.1% SPC and 15.6% CBM. CMM diet contained 15.4% CGM and 12.1% MM. CCM diet contained 15.4% CGM and 15.6% CBM. MCM diet contained 12.1% MM and 15.6% CBM. The 12th diet (MIX) was formulated by including SPC, CGM, MM, and CBM at 7.55%, 7.70%, 6.03%, and 7.80%. respectively. All dry ingredients were thoroughly mixed with oil and 30% distilled water to make a dough. Then, the dough was passed through a mincing machine (SP-50, Gum Gang Engineering, Daegu, Korea) fitted with 3 mm die, collected 3 mm diameter pellets to metal trays, crushed into 5–7 mm size, and dried at 30°C for 12 hr to prepare as sinking dry pellets. Dry diets were stored at −20°C until use. Proximate composition of protein sources is provided in Table 2. Fatty acids and amino acids composition of protein sources and experimental diets are presented in Tables 3–6.

### 2.2. Feeding Trial and Experimental Conditions

Red seabreams in juvenile stage were obtained from a private hatchery (Tongyeong, Korea) and transferred to the fish and shellfish holding facilities at the Gangneung–Wonju National University, Marine Biology Center. Fish were acclimatized to experimental facilities and conditions for 2 weeks while feeding a commercial diet (51% protein, 12% lipid; Aller Aqua Co., Ltd., Qingdao, China). After 2 weeks acclimation period, red seabream, averaging 50.2 ± 0.1 g, was stocked at a density of 15 fish per tank in 36 fiberglass tanks having a 300 L capacity. Tanks were in a flow-through system supplied with continuous seawater flow and aerated with sandstones. Each tank was randomly assigned to one of the three replicates of 12 dietary treatments. Fish were fed one of the experimental diets to apparent satiation (twice a day, 09:00 and 17:00 hr) for 12 weeks. The uneaten feed was collected 30 min after feeding, dried in an oven at 105°C for 6 hr and reweighed to determine precise feed intake. The photoperiod was controlled to closely resemble the natural day length of the season. Water temperature (18.9 ± 0.3°C) was monitored every day and the other quality values including dissolved oxygen (7.72 ± 0.5 mg/L; Mean ± SE), pH (7.56 ± 0.3), and salinity (33.1 ± 0.4 ppt) were monitored throughout the feeding trial.

### 2.3. Sample Collection and Analyses

At the end of the feeding trial, all remaining fish in each experimental tank were starved for 18 hr, counted, and bulk weighed for the calculation of survival rates, growth performance, and feed efficiency including weight gain (WG), daily feed intake (DFI), feed efficiency (FE), and protein efficiency ratio (PER). Six fish were randomly sampled from each tank and anesthetized with 2-phenoxyethanol (200 ppm). Blood samples were collected from three fish per tank using heparinized syringes to separate plasma for biochemical analyses and from the other three fish using nonheparinized syringes to separate serum samples for immune parameter analyses. Both plasma and serum were separated by centrifugation at 5,000 *g* for 10 min and stored at −70°C. Blood samples were allowed to clot at room temperature for 30 min prior to separating serum samples. After blood sampling, fish were stored frozen at −20°C for proximate analyses. The remaining fish in each tank were killed, the total length of fish was measured to the nearest 0.1 mm and their viscera and livers were dissected and weighed to determine hepatosomatic (HSI) and viscerosomatic indices (VSI). The fillet of fish was also sampled for the analysis of proximate composition, amino acids, and fatty acid levels.

Moisture and ash levels of the experimental ingredients, diets and fillet samples of fish were analyzed according to standard methods [33]. Crude protein was measured using an automatic Kjeltec Analyzer (Buchi, Flawil, Switzerland) and crude lipid was determined using a Soxhlet extractor (VELP Scientifica, Milano, Italy). Fatty acid profiles of the protein sources, experimental diets, and fillet were analyzed using a gas chromatographic method as mentioned by Sankian et al. [34]. An automatic amino acid analyzer (Hitachi, Tokyo, Japan) was used to estimate the amino acid composition of the protein sources, experimental diets, and fillet. Serum lysozyme activity was measured based on a turbidimetric technique using lyophilized *Micrococcus lysodeikticus* (Sigma, St. Louis, MO, USA) as a substrate [28]. Serum superoxide dismutase activity (SOD) was measured using a Colorimetric Assay Kit (19,160, Sigma, USA). An automated blood analyzer (FUJI DRI-CHEM NX500i, FUJiFILM Corporation Tokyo, Japan) was used for measure plasma glutamic–oxaloacetic transaminase (GOT), glutamate pyruvate transaminase (GPT), total bilirubin (TBIL), total protein (TP), albumin (ALB), glucose (GLU), ammonia (NH_3_), amylase (AMYL), and total cholesterol (TCHO) levels with dri-chem slides (reference code: 3150, 3250, 2150, 1850, 2050, 1050, 1850, 4350, and 1450, respectively) purchased from FUJiFILM Co., Japan.

### 2.4. Statistical Analysis

Data were subjected to one-way analysis of variance. Then, Duncan's [35] multiple range test was used to determine the significance of differences in the mean effects of diets, using SPSS version 20.0 (SPSS Inc., Chicago, IL). Shapiro–Wilk's and Levene's tests were applied to verify whether the normality and homogeneity of variances are met. Statistical significance was determined at *P* < 0.05. Data were presented as mean ± standard error (SE). Percentage data were arcsine transformed before statistical analysis.

## 3. Results

Growth performance, feed utilization, and biometric parameters of red seabream are shown in Table 7. Final body weight (FBW) and WG of red seabream were significantly improved by MM and MCM diets compared to that of fish fed CG, SCG, CMM, and CCM diets. Interestingly, the FBW and WG results of CON, SPC, CM, SMM, SCM, and MIX groups were comparable with the MM and MCM groups. The results observed in CON, SMM, and SCM groups were significantly higher compared to that of CG, CMM, and CCM groups. CM and MIX groups exhibited significantly higher FBW and WG compared to CG and CMM groups. FE was significantly higher in MM group compared to that of fish fed SPC, CG, SGC, and CCM diets. Fish fed MCM diet exhibited significantly higher FE compared to CG and SCG groups. However, FE of CON, CM, SMM, SCM, CMM, and MIX groups were comparable with all the other groups. DFI, survival, and biometric parameters were not significantly affected by FM replacement or inclusion of alternative ingredients in diets.

Proximate composition of red seabream whole-body and fillet were presented in Table 8. FM replacement or inclusion of alternative ingredients in diet did not significantly affect the proximate composition of fish whole-body or fillets.

Fillet fatty acid profiles of red seabream are presented in Table 9. Linolenic acid (C18:2*n*−6) level of fish fed CG diet was significantly higher than CON, MM, CM, SCM, CCM, and MCM groups. CM group exhibited significantly lower linolenic acid compared to fish fed SPC, CG, SCG, and CMM diets. However, the deposition of other fatty acids was not significantly influenced by alternative ingredients.

Fillet essential amino acid levels fish fed experimental diets are presented in Table 10. After 12 weeks feeding trial, fillet amino acid compositions were not significantly affected by the ingredients.

Biochemical parameter in red seabream fed experimental diets are presented in Table 11. The tested parameters were not significantly affected by the experimental diets. However, the lysozyme activity of serum was significantly higher in fish fed MCM and MIX diets compared to that of the other groups. Serum SOD activity was not significantly affected by the experimental diets (Table 12).

## 4. Discussion

Growth performance of red seabream was significantly improved by MM and MCM feed although DFI of fish groups was not significantly different after 12 weeks of feeding trial. Therefore, it is obvious that efficiency of feed was improved by MM alone or together with CBM. Experimental diets were formulated to be isonitrogenous and isolipidic resulting in only slight variation in total amino acid and fatty acid compositions although total omega-3, omega-6, and HUFA levels were lower in all low-FM diets (Table 5). Therefore, growth performance of red seabream fed SPC, MM, CM, SMM, SCM, MCM, and MIX diets were not significantly retarded due to the reduced levels of dietary fatty acids after 12 weeks. Growth performance of fish fed SPC, SMM, and SCM diets also exhibited significantly comparable results to CON, MM, and MCM groups. Only the diets containing CG resulted in significantly lower growth performance indicating that GC was not a suitable protein source to include in high proportion as a FM replacer in low-FM diets for red seabream.

MM used in the present study was prepared using meat byproducts without bones. The protein level was higher in MM (84%) as a result of its protein-rich raw material. MM also exhibited high-pepsin digestibility which was comparable to sardine FM and SPC (Table 2). Rahman et al. [15] reported that nutrient digestibility of MM (84% CP) was higher in olive flounder compared to different conventional FM. Therefore, nutrient digestibility of diets might be improved by MM in red seabream to observe higher growth performance and feed utilization. Moreover, MM contained the highest crude protein level compared to the other ingredients resulting in a low-inclusion level in diets compared to tested ingredients in other diets because the inclusion level of each ingredient was decided to compensate for reduced protein level after the FM replacement. Therefore, MM-containing diets were formulated to contain high-wheat flour levels. Wheat flour contains a high level of starch which can easily be digested in the red seabream [36]. They also observed that starch can improve efficiency of red seabream diets compared to other carbohydrate sources. Therefore, high-protein level of MM might influence indirectly to improve FE in the present study. Supportively, DFI of fish was not significantly different among treatments while FE was higher in fish-fed diets containing MM except for CMM. Animal protein sources contain a more balanced nutrient profile compared to plant protein sources [15, 37]. Lu et al. [38] observed that growth performance and FE of rainbow trout were increased by animal protein sources more efficiently than plant protein sources. Tidwell et al. [39] also reported a similar trend in largemouth bass (*Micropterus salmonids*) fed animal or plant protein sources. They suggested that reduced feed intake due to low palatability of plant protein sources was the main reason for the retarded growth performance. In the present study, DFI was not significantly changed with diets indicating that feed palatability was not affected by plant protein sources during the trial period. Murashita et al. [40] found that the animal proteins and soybean meal were digestive stimulants in red seabream compared to CG and SPC. Supportively, digestive enzyme activities such as alkaline phosphatase, lipase, and leucine aminopeptidase in gilthead seabream were not decelerated by dietary CBM even after replacing 100% FM [21]. Therefore, we assumed that highly digestible nutrient in MM and CBM was a reason for high-growth rates observed in MM, CM, SMM, MCM, and MIX groups compared to other diets. Nutrient digestibility of these diets and each ingredient should be estimated in red seabream to elucidate the assumption in future studies.

Plant protein sources were also reportedly effective in FM replacement in red seabream diet [5, 30]. Especially, several studies revealed that addition of the amino acids or functional ingredients such as taurine, methionine, and lysine improve the efficiency of red seabream diets containing a high level of plant protein [6, 7, 30]. In the present study, SPC and GC-containing diets except for SMM and SCM showed lower growth performance compared to the control. Dietary CG was reported to decrease protein, lipid, and amino acid digestibility in turbot (*Psetta maxima*), when they were fed a diet containing 20% CG [41]. However, they observed no significant effects on growth performance and FE after feeding 20% CG although PER was significantly reduced. Similar results were observed in sunshine bass (*Morone chrysops* × *M. saxatilis*) and juvenile Ussuri catfish (*Pseudobagrus ussuriensis*) when they were fed high level of CG [42, 43]. Diet digestibility, digestive enzyme activities, and gene expression were reduced in these species. Therefore, it seems like a high level of CG can adversely affect fish performance. However, optimum FM replacement of CG in red seabream diet was not evaluated to the best of our knowledge. The results of present study indicate that optimum FM replacement level of CG should be obviously lower than 30% because of the lower performance observed in the group. Moreover, red seabream fed CMM and CCM diets also exhibited significantly lower growth performance although those diets contained approximately 15% CG and either MM or CM. It is indicated that GC supplementation level in low-FM diets should also be lower than 15% when a single protein source was used substitute FM with GC. MIX group exhibited comparable growth performance to CON group indicating 7.5% CG as an effective level for red seabream diet when FM was replaced using a mixture of alternative protein ingredients. SMM and SCM diets also improved the growth performance of red seabream showing comparable values to CON diet. SPC was reported as an effective FM replacer in feed for several carnivorous fish species including red seabream [5]. Especially, SPC can be successfully used as FM replacer when functional ingredients and alternative protein sources were included in feed containing low-FM and high-SPC levels [6, 8, 29]. According to those studies, SPC was unable to restore red seabream growth when 50% of FM was replaced in feed with SPC. Alternative ingredients improved feed intake and nutrient digestibility in diets containing high-SPC levels. Accordingly, nutrient digestibility of SMM and SCM diets might be improved in the present study due to the effects of MM and CM. Therefore, nutrient digestibility of feed containing SPC with animal protein sources should be investigated in future studies to prove the assumption. Moreover, the low-inclusion level of SPC might also be beneficial on fish growth as observed in the aforementioned studies.

Fillet proximate, fatty acid, and amino acid compositions were not significantly affected by single or combinations of protein sources in diets except for linoleic acid levels. Red seabream fed feeds containing plant protein sources exhibited high-linoleic acid levels compared to those fed diets containing only animal protein sources. Linoleic acid level in SPC and CG is considerably higher than the other protein sources (Table 3). Therefore, observed changes in fatty acid profile were expected in fish fed plant protein sources because of dietary composition. However, the slight differences in fatty acid and amino acid levels in each diet were not reflected in fillet composition indicating that fillet quality was not sorely affected by dietary ingredients. In contrast, fillet and whole-body fatty acid levels were significantly affected in red seabream fed diets containing different fatty acid levels [44–46]. Whole-body proximate composition of red seabream was significantly changed by diets in these three studies. Growth performance was also not significantly changed except in a non-FM and nonfish oil group evaluated by Seong et al. [46]. In the present study, growth performance was significantly changed although whole-body or fillet compositions were not significantly affected by experimental diets. Therefore, we assumed that the fish growth was limited to available nutrients in ingredients instead of changing fillet composition to reflect diet composition in the present study. However, the discrepancy should be evaluated in future studies.

Plasma biochemical parameters of red seabream were not significantly affected by the experimental diets (Table 11). According to previous reports, biochemical indices of red seabream were significantly affected due to different reasons. Zaineldin et al. [47] mentioned that an increased number of immune cells in blood was the reason for increased biochemical measurements in their study. Kim and Kang [48] indicated that environmental stress was a reason for increased biochemical parameters in red seabream exposed to waterborne selenium. Main dietary protein sources also affected plasma biochemical indices in red seabream [49]. Therefore, blood biochemical parameters can be considered as indicators of environmental impact health status and feed quality of red seabream. Results of present study indicate that the FM replacement or inclusion of protein sources was not adversely influenced on biochemical composition of red seabream blood within 12 weeks feeding period. However, effects of these protein sources on biochemical parameters of red seabream should be deeply investigated in future studies.

Serum lysozyme activity was significantly increased in red seabream fed MCM and MIX diets although SOD activity was not significantly affected by diets. Lysozyme activity of red seabream is increased when their diet contains favorable nutrition levels. High-lysozyme activity is usually observed in fish groups having high-growth performance and feed utilization [30, 47, 50]. Saurabh and Sahoo [51] also reported that the lysozyme activity of fish can be boosted by maintaining proper nutrient status. Accordingly, results in the present study indicate that the fish in MCM and MIX groups had suitable nutrient levels for red seabream compared to the other diets. However, growth performance in MM, CON, SPC, CM, SMM, and SCM groups were also significantly higher than other groups and comparable with MCM and MIX groups indicating that other phenomena can also affect the lysozyme activity of red seabream. High levels of dietary plant protein also resulted in reduced lysozyme activity in red seabream [28, 49] although MIX group in the present study exhibited high-lysozyme activity while containing approximately 15% of SPC and CG in the diet. Therefore, we assumed that matching nutrient content for red seabream was provided by MCM and MIX diets compared to other diets used in the present study. Several studies reported that diets formulated to contain a combination of protein ingredients were more efficient in improving fish performance than diets containing fewer protein ingredients [16, 52]. Therefore, different combinations of these ingredients should be evaluated in future studies to find proper dietary inclusion levels for improving both innate immunity and growth performance of red seabream.

In summary, the results of the present study clearly indicated that a high level of CG in low-FM diets reduces the growth performance and feed utilization of red seabream. SPC can be efficiently used as a protein ingredient when mixed with other alternative protein sources to replace FM. MM and CBM are suitable FM replacers in red seabream diets. Especially, a mixture of both MM and CBM seems to be more efficient than a single ingredient in diets. Therefore, we concluded that MCM and MIX diets are more efficient in replacing FM from red seabream diet compared to other diets tested in the present study. Future studies should be conducted to evaluate the effects of different levels of ingredient mixtures used in both MCM and MIX diets for red seabream.

## Figures and Tables

**Table 1:** Formulation and proximate compositions of the experimental diets (% dry matter).

	Experimental diets											
	CON	SPC	CG	MM	CM	SCG	SMM	SCM	CMM	CCM	MCM	MIX
Tuna-byproduct meal	30.0	15.0	15.0	15.0	15.0	15.0	15.0	15.0	15.0	15.0	15.0	15.0
Pollock fishmeal	15.0	7.50	7.50	7.50	7.50	7.50	7.50	7.50	7.50	7.50	7.50	7.50
Sardine fishmeal	15.0	7.50	7.50	7.50	7.50	7.50	7.50	7.50	7.50	7.50	7.50	7.50
Soy protein concentrate		30.2				15.1	15.1	15.1				7.55
Corn gluten meal			30.8			15.4			15.4	15.4		7.70
Meat meal				24.1			12.1		12.1		12.1	6.03
Chicken byproduct meal					31.2			15.6		15.6	15.6	7.8
Squid liver powder	3.00	3.00	3.00	3.00	3.00	3.00	3.00	3.00	3.00	3.00	3.00	3.00
Wheat flour	28.18	25.98	24.98	34.08	28.98	25.68	30.03	27.48	29.73	27.18	31.53	28.60
Mono calcium phosphate	0.20	0.20	0.20	0.20	0.20	0.20	0.20	0.20	0.20	0.20	0.20	0.20
Lecithin	1.00	1.00	1.00	1.00	1.00	1.00	1.00	1.00	1.00	1.00	1.00	1.00
Fish oil	6.00	8.00	8.40	6.00	4.00	8.00	7.00	6.00	7.00	6.00	5.00	6.50
Choline	0.12	0.12	0.12	0.12	0.12	0.12	0.12	0.12	0.12	0.12	0.12	0.12
Vitamin C	0.50	0.50	0.50	0.50	0.50	0.50	0.50	0.50	0.50	0.50	0.50	0.50
Vitamin premix^1^	0.50	0.50	0.50	0.50	0.50	0.50	0.50	0.50	0.50	0.50	0.50	0.50
Mineral premix^2^	0.50	0.50	0.50	0.50	0.50	0.50	0.50	0.50	0.50	0.50	0.50	0.50
Proximate composition												
Crude protein	47.5	46.2	47.1	47.2	46.9	46.3	48.2	46.8	47.9	46.6	48.2	47.5.
Crude lipid	14.4	13.1	14.1	14.1	13.5	14.3	13.6	13.5	13.5	13.5	13.6	14.1
Ash	12.6	8.40	7.40	8.60	14.0	7.30	8.80	11.2	7.90	10.7	11.3	9.60

*Note*: ^1^Vitamin mixture composition (unit/kg mix): ascorbic acid, 6,400 mg; tocopherol acetate, 37,500 mg; thiamin nitrate, 5,000 mg; riboflavin, 10,000 mg; pyridoxine hydrochloride, 5,000 mg; nicotinic acid, 37,500 mg; Ca-D-pantothenate, 17,500 mg; inositol, 75,000 mg; biotin, 50 mg; folic acid, 2,500 mg; menadione sodium bisulfite, 2,500 mg; retinol acetate, 5,000,000 IU; cholecalciferol, 1,000,000 IU; cyanocobalamin, 25 mg; riboflavin, 10,000 mg, ^2^Mineral mixture composition (g/kg mix); ferrous fumarate, 12.5; manganese sulfate, 11.3, ferrous sulfate, 20; cupric sulfate, 1.25; cobaltous sulfate, 0.75; zinc sulfate, 13.75; calcium iodate, 0.75; magnesium sulfate, 80.2; aluminum hydroxide, 0.75. CON, 60% FM; SPC, 30% FM with SPC; CG, 30% FM with CG; MM, 30% FM with MM; CM, 30% FM with CM; SCG, 30% FM with SPC and GC; SMM, 30% FM with SPC and MM; SCM, 30% FM with SPC and CM; CMM, 30% FM CG and MM; CCM, 30% FM with CG and CM; MCM, 30% FM with MM and CM; MIX, 30% FM with SPC, GC, MM, and CM.

**Table 2:** Proximate composition of the various animal and plant feed ingredients (% dry matter).

Protein source	Dry matter	Crude protein	Crude lipid	Ash	Calcium^*∗*^	Phosphorus^*∗*^	Pepsin digestibility^*∗*^
Tuna byproduct meal^1^	93.8	64.0	6.40	20.7	4.27	2.16	92.9
Sardine fish meal^2^	93.8	71.1	8.80	15.1	3.52	2.30	96.3
Pollock fish meal^3^	93.0	71.7	8.30	17.0	4.42	2.60	95.9
Soy protein concentrate (SPC)^4^	96.2	67.2	1.00	6.30	0.62	0.68	96.2
Corn gluten (CG)^5^	92.8	65.9	0.10	1.10	0.92	0.44	94.0
Meat meal (MM)^6^	97.1	84.2	9.50	5.80	1.30	0.89	96.5
Chicken byproduct meal (CM)^7^	98.1	62.5	11.1	20.9	4.59	2.66	89.8

*Note*: ^1^Woojin feed Ind. Co. Ltd., Incheon, South Korea; ^2^Cesmec Ltda., Santiago, Chile; ^3^Kodiak fish meal Company, Alaska, USA; ^4^Solae, St. Louis, USA; ^5^The Feed Co., Goyang, South Korea; ^6^Hwasong industrial Co. Ltd., Seogwipo, South Korea; ^7^Woosin Food Co. Ltd., Pocheon, South Korea.  ^*∗*^Obtained from product technical data sheets.

**Table 3:** Fatty acid profiles and lipid nutritional quality indices of ingredients (% of total fatty acids).

	Ingredients						
	Tuna byproduct meal	Sardine fish meal	Pollock fish meal	Soy protein concentrate	Corn gluten	Meat meal	Chicken byproduct meal
C14:0	4.10	5.80	4.20	ND^1^	0.20	2.30	0.70
C16:0	23.3	20.6	17.7	10.2	13.8	27.7	23.7
C18:0	7.10	5.40	4.10	3.90	ND	14.8	8.00
*Σ*SFA^2^	39.2	34.7	27.9	16.0	16.0	47.6	34.5
C16:1*n*−7	6.10	6.60	6.30	ND	0.20	3.10	3.60
C18:1*n*−9	20.6	15.0	19.6	58.4	25.0	42.0	45.4
*Σ*MUFA^3^	34.6	23.7	39.5	58.8	25.7	48.0	50.6
C18:2*n*−6	2.70	5.70	2.30	20.5	55.5	3.60	13.4
*Σ*n6 FA^4^	3.90	7.00	3.60	20.5	56.0	4.40	14.3
C18:3*n*−3	1.40	1.00	1.40	4.70	2.30	ND	0.60
C20:5*n*−3	5.50	16.6	14.5	ND	ND	ND	ND
C22:6*n*−3	15.1	17.00	13.1	ND	ND	ND	ND
*Σn*3 FA^5^	22.3	34.5	29.0	4.70	2.30	ND	0.60
HUFA^6^	21.0	33.8	27.7	ND	0.20	0.20	0.20
*Σn*3/*Σn*6	5.70	4.90	8.00	0.20	ND	ND	ND

*Note*: ^1^Not detected; ^2^saturated fatty acids; ^3^monounsaturated fatty acids; ^4^*n*−6 polyunsaturated fatty acids; ^5^*n*−3 polyunsaturated fatty acids; ^6^highly unsaturated fatty acids; the fatty acids C12:0, C13:0, C14:1*n*−5, C15:0, C16:1*n*−9, C17:0, C17:1, C18:1*n*−7, C18:3*n*−6; C20:0; C20:1*n*−9; C20:2*n*−6, C20:3*n*−6, C20:3*n*−3, C20:4*n*−6, C20:4*n*−3, C22:0, C22:1*n*−9, C22:2*n*−6, C22:5*n*−3, and C24:0 in percentage ≤ 1%, were also detected and used to calculate the fatty acid groups.

**Table 4:** Essential amino acid composition of ingredients (% of protein).

	Ingredients						
	Tuna byproduct meal	Sardine fish meal	Pollock fish meal	Soy protein concentrate	Corn gluten	Meat meal	Chicken byproduct meal
Arg	6.2	6.3	6.8	7.3	3.0	7.6	7.4
His	3.7	3.0	2.4	2.9	2.1	2.2	2.5
Ile	3.9	4.2	3.7	4.0	3.2	2.4	2.1
Leu	7.3	8.0	7.3	7.7	15	5.4	6.7
Lys	7.8	9.0	8.1	6.6	2.0	5.8	6.5
Met + Cys	4.6	4.7	4.7	2.9	4.2	2.7	3.1
Phe	3.8	4.2	3.6	4.8	5.4	2.9	3.9
Thr	4.6	4.8	4.5	4.1	3.2	3.2	4.0
Val	5.4	5.4	4.9	4.7	4.2	4.1	3.4

**Table 5:** Fatty acid profiles and lipid nutritional quality indices of experimental diets (% of total fatty acids).

	Experimental diets												
	CON	SPC	CG	MM	CM	SCG	SMM	SCM	CMM	CCM	MCM	MIX
C14:0	2.40	1.80	1.60	2.00	1.70	1.70	1.90	1.70	1.80	1.60	1.80	1.80
C16:0	19.3	17.4	16.9	20.8	21.2	17.3	18.8	19.4	18.5	19.1	20.6	18.9
C18:0	7.20	6.80	6.20	8.80	8.00	6.60	7.60	7.40	7.20	7.10	8.20	7.40
*Σ*SFA^1^	30.8	27.6	26.3	33.3	32.9	27.1	29.9	30.1	29.1	29.5	32.4	29.7
C16:1*n*−7	3.60	2.80	2.50	3.10	3.40	2.60	2.90	2.90	2.70	2.90	3.20	2.90
C18:1*n*−9	22.8	24.5	24.2	25.9	28.9	24.4	25.3	26.7	25.1	26.2	27.4	25.9
*Σ*MUFA^2^	29.5	29.9	29.0	31.8	34.7	29.5	30.9	32.1	30.4	31.6	33.2	31.4
C18:2*n*−6	24.6	30.0	33.5	24.0	22.6	31.7	27.6	26.6	29.6	28.3	24.0	27.4
*Σn*6 FA^3^	25.5	31.0	34.4	24.9	23.4	32.6	28.5	27.5	30.4	29.1	24.8	28.2
C18:3*n*−3	3.70	4.40	4.20	3.30	2.80	4.30	4.00	3.70	3.90	3.50	3.20	3.70
C20:5*n*−3	4.20	2.90	2.50	2.70	2.50	2.70	2.70	2.70	2.50	2.60	2.50	2.70
C22:6*n*−3	6.30	4.20	3.60	4.00	3.60	3.80	4.00	3.90	3.60	3.70	3.70	4.10
*Σn3* FA^4^	14.2	11.5	10.4	10.0	9.0	10.8	10.7	10.3	10.1	9.80	9.50	10.6
HUFA^5^	10.7	7.30	6.30	6.90	6.30	6.80	6.90	6.90	6.30	6.40	6.40	7.00
*Σn*3/*Σn*6	0.60	0.40	0.30	0.40	0.40	0.30	0.40	0.40	0.30	0.30	0.40	0.40

*Note*: ^1^Saturated fatty acids; ^2^monounsaturated fatty acids; ^3^*n*-6 polyunsaturated fatty acids; ^4^*n*-3 polyunsaturated fatty acids; ^5^highly unsaturated fatty acids. The fatty acids C12:0, C13:0, C14:1*n*−5, C15:0, C16:1*n*−9, C17:0, C17:1, C18:1*n*−7, C18:3*n*−6; C20:0; C20:1*n*−9; C20:2*n*−6, C20:3*n*−6, C20:3*n*−3, C20:4*n*−6, C20:4*n*−3, C22:0, C22:1*n*−9, C22:2*n*−6, C22:5*n*−3, and C24:0 in percentage ≤ 1%, were also detected and used to calculate the fatty acid groups. CON, 60% FM; SPC, 30% FM with SPC; CG, 30% FM with CG; MM, 30% FM with MM; CM, 30% FM with CM; SCG, 30% FM with SPC and GC; SMM, 30% FM with SPC and MM; SCM, 30% FM with SPC and CM; CMM, 30% FM with CG and MM; CCM, 30% FM with CG and CM; MCM, 30% FM with MM and CM; MIX, 30% FM with SPC, GC, MM, and CM.

**Table 6:** Essential amino acid composition of experimental diets (% of protein).

	Experimental diets											
	CON	SPC	CG	MM	CM	SCG	SMM	SCM	CMM	CCM	MCM	MIX
Arg	5.9	6.2	4.4	6.6	6.5	5.6	6.7	6.6	5.6	5.5	6.4	6.0
His	2.9	3.0	2.6	2.7	2.8	2.7	2.8	2.9	2.6	2.6	2.7	2.7
Ile	3.9	3.9	3.6	3.3	3.7	3.8	3.7	3.9	3.4	3.7	3.5	3.5
Leu	7.3	7.2	10.9	6.4	7.1	9.5	7.0	7.3	8.9	9.4	6.8	8.0
Lys	7.4	7.0	4.6	6.5	7.0	5.6	6.6	7.0	5.4	5.8	6.6	6.3
Met + Cys	4.4	4.0	4.4	3.8	4.5	3.9	3.8	4.0	4.1	4.0	4.4	3.8
Phe	4.0	4.3	4.6	3.6	3.8	4.5	3.9	4.1	4.1	4.3	3.7	3.9
Thr	4.5	4.3	3.8	3.9	4.3	4.0	4.0	4.3	3.8	4.0	4.0	4.0
Val	5.4	5.2	4.9	4.9	5.2	5.0	5.0	5.1	4.8	5.0	4.9	5.1

CON, 60% FM; SPC, 30% FM with SPC; CG, 30% FM with CG; MM, 30% FM with MM; CM, 30% FM with CM; SCG, 30% FM with SPC and GC; SMM, 30% FM with SPC and MM; SCM, 30% FM with SPC and CM; CMM, 30% FM with CG and MM; CCM, 30% FM with CG and CM; MCM, 30% FM with MM and CM; MIX, 30% FM with SPC, GC, MM, and CM.

**Table 7:** Growth performance, feed utilization efficiency, and biometric parameters of red seabream fed the experimental diets for 12 weeks.

	Experimental diets											
	CON	SPC	CG	MM	CM	SCG	SMM	SCM	CMM	CCM	MCM	MIX
FBW^1^	146 ± 2.39^ab^	136 ± 5.89^abcd^	129 ± 2.51^d^	148 ± 0.97^a^	145 ± 1.25^abc^	134 ± 3.22^bcd^	146 ± 1.16^ab^	146 ± 2.46^ab^	129 ± 7.04^d^	133 ± 6.97^cd^	148 ± 3.15^a^	142 ± 2.22^abc^
WG^2^	191 ± 4.8^ab^	171 ± 11^abcd^	157 ± 5.0^d^	195 ± 1.7^a^	188 ± 2.5^abc^	167 ± 6.4^bcd^	191 ± 2.2^ab^	192 ± 5.2^ab^	157 ± 14^d^	165 ± 14^cd^	196 ± 6.2^a^	183 ± 4.4^abc^
DFI^3^	1.23 ± 0.0	1.20 ± 0.1	1.22 ± 0.1	1.15 ± 0.0	1.26 ± 0.0	1.24 ± 0.1	1.21 ± 0.0	1.26 ± 0.0	1.15 ± 0.1	1.22 ± 0.0	1.18 ± 0.0	1.21 ± 0.1
FE^4^	92.4 ± 2.8^abc^	88.9 ± 1.4^bc^	83.8 ± 2.6^c^	102 ± 3.0^a^	91.6 ± 2.0^abc^	87.0 ± 5.2^c^	95.1 ± 0.8^abc^	92.8 ± 2.8^abc^	89.4 ± 0.3^abc^	87.6 ± 1.5^bc^	100 ± 2.4^ab^	92.4 ± 2.6^abc^
Sur^5^	95.6 ± 2.2	95.6 ± 2.2	95.6 ± 2.2	100 ± 0.0	100 ± 0.0	97.8 ± 2.2	97.8 ± 2.2	100 ± 0.0	97.8 ± 2.2	100 ± 0.0	100 ± 0.0	95.6 ± 2.2
CF^6^	1.79 ± 0.07	1.53 ± 0.05	1.67 ± 0.09	1.65 ± 0.02	1.80 ± 0.10	1.65 ± 0.03	1.67 ± 0.03	1.61 ± 0.03	1.65 ± 0.01	1.65 ± 0.01	1.71 ± 0.03	1.67 ± 0.04
HSI^7^	1.64 ± 0.04	1.34 ± 0.14	1.64 ± 0.11	1.76 ± 0.14	1.55 ± 0.07	1.43 ± 0.05	1.54 ± 0.24	1.37 ± 0.11	1.39 ± 0.15	1.68 ± 0.09	1.58 ± 0.11	1.46 ± 0.08
VSI^8^	7.17 ± 0.29	6.43 ± 0.50	7.02 ± 0.24	7.34 ± 0.78	7.27 ± 0.05	6.69 ± 0.21	6.67 ± 0.55	6.68 ± 0.17	5.98 ± 0.15	6.34 ± 0.58	6.85 ± 0.52	6.76 ± 0.34

*Note*: Values are mean of triplicate groups and presented as mean ± SE. ^a,b,c,d^Different superscripts in the same row indicate significant differences between the means by ANOVA at *P* < 0.05. The lack of superscripts in the same row indicates no significant difference between the means by ANOVA at *P* > 0.05. ^1^Final body weight; ^2^weight gain = (final body weight-initial body weight) × 100/initial body weight; ^3^daily feed intake (g/fish) = (dry feed consumed (g)/fish number)/trial period (days). ^4^Feed efficiency = wet weight gain × 100/feed intake; ^5^survival (%) = (final number of fish/initial number of fish) × 100; ^6^Condition factor (%) = (wet weight of fish/(length of fish)3) × 100; ^7^hepatosomatic index (%) = (wet weight of liver/wet weight of fish) × 100; ^8^viscerasomatic index (%) = (wet weight of viscera/wet weight of fish) × 100. CON, 60% FM; SPC, 30% FM with SPC; CG, 30% FM with CG; MM, 30% FM with MM; CM, 30% FM with CM; SCG, 30% FM with SPC and GC; SMM, 30% FM with SPC and MM; SCM, 30% FM with SPC and CM; CMM, 30% FM CG and MM; CCM, 30% FM with CG and CM; MCM, 30% FM with MM and CM; MIX, 30% FM with SPC, GC, MM, and CM.

**Table 8:** Proximate composition of fillet and whole-body of red seabream fed the experimental diets for 12 weeks (% on wet matter basis).

	Experimental diets											
	CON	SPC	CG	MM	CM	SCG	SMM	SCM	CMM	CCM	MCM	MIX
Fillet												
Moisture	72.6 ± 1.2	75.5 ± 0.8	74.4 ± 1.8	72.4 ± 1.9	73.8 ± 1.5	74.2 ± 0.5	73.3 ± 0.6	74.8 ± 0.6	73.2 ± 0.2	73.4 ± 0.4	74.6 ± 0.3	74.0 ± 0.4
Crude protein	23.1 ± 0.4	21.2 ± 0.3	21.7 ± 0.7	22.4 ± 0.9	22.2 ± 0.7	21.9 ± 0.3	22.1 ± 1.5	22.2 ± 0.2	21.4 ± 0.2	21.5 ± 0.3	22.8 ± 0.7	21.9 ± 0.8
Crude lipid	1.3 ± 0.3	1.2 ± 0.3	1.4 ± 0.2	1.4 ± 0.2	1.1 ± 0.4	1.4 ± 0.3	1.2 ± 0.2	1.2 ± 0.2	1.3 ± 0.2	1.3 ± 0.3	1.2 ± 0.1	1.3 ± 0.3
Ash	1.9 ± 0.1	1.9 ± 0.1	1.9 ± 0.2	1.8 ± 0.1	1.8 ± 0.1	1.8 ± 0.1	1.7 ± 0.1	1.7 ± 0.1	1.5 ± 0.2	1.7 ± 0.2	1.7 ± 0.2	1.9 ± 0.1
Whole body												
Moisture	65.4 ± 0.3	67.1 ± 1.2	67.7 ± 0.5	66.3 ± 0.8	66.0 ± 0.2	66.7 ± 0.6	65.9 ± 0.8	66.9 ± 0.7	67.1 ± 0.5	67.1 ± 0.7	65.7 ± 2.2	69.5 ± 0.8
Crude protein	18.6 ± 0.1	18.2 ± 0.3	17.9 ± 0.4	17.2 ± 0.7	17.2 ± 0.5	18.1 ± 0.5	17.3 ± 0.3	17.5 ± 0.3	17.9 ± 0.4	17.9 ± 0.1	18.7 ± 0.2	18.6 ± 0.1
Crude lipid	7.2 ± 0.5	7.5 ± 0.1	8.2 ± 0.7	7.3 ± 0.7	6.5 ± 0.2	8.7 ± 0.4	7.9 ± 1.4	6.9 ± 0.8	7.4 ± 0.2	7.7 ± 1.4	7.1 ± 0.4	8.2 ± 1.4
Ash	7.0 ± 0.1	6.1 ± 0.2	5.6 ± 1.4	5.6 ± 0.4	7.2 ± 0.5	5.8 ± 0.3	7.3 ± 0.4	6.4 ± 0.2	6.3 ± 0.4	6.9 ± 0.5	7.0 ± 0.8	5.8 ± 0.4

*Note*: Values are means of triplicate groups and are presented as mean ± SE. CON, 60% FM; SPC, 30% FM with SPC; CG, 30% FM with CG; MM, 30% FM with MM; CM, 30% FM with CM; SCG, 30% FM with SPC and GC; SMM, 30% FM with SPC and MM; SCM, 30% FM with SPC and CM; CMM, 30% FM with CG and MM; CCM, 30% FM with CG and CM; MCM, 30% FM with MM and CM; MIX, 30% FM with SPC, GC, MM, and CM.

**Table 9:** Fillet fatty acid profiles and lipid nutritional quality indices of red seabream fed the experimental diets for 12 weeks (% of total fatty acids).

	Experimental diets												
	CON	SPC	CG	MM	CM	SCG	SMM	SCM	CMM	CCM	MCM	MIX
C14:0	2.4 ± 0.0	1.8 ± 0.2	2.0 ± 0.1	1.7 ± 0.3	1.9 ± 0.1	2.2 ± 0.2	1.9 ± 0.0	1.7 ± 0.1	1.9 ± 0.1	1.9 ± 0.1	2.1 ± 0.3	2.1 ± 0.2
C16:0	18.4 ± 0.3	18.2 ± 0.3	18.4 ± 0.3	19.1 ± 0.1	19.1 ± 0.1	17.7 ± 0.5	18.3 ± 0.1	18.8 ± 0.0	18.0 ± 0.1	18.5 ± 0.2	18.8 ± 0.3	17.7 ± 0.5
C18:0	6.5 ± 0.1	7.1 ± 0.2	7.0 ± 0.1	7.4 ± 0.4	7.1 ± 0.0	6.7 ± 0.3	6.8 ± 0.2	7.3 ± 0.2	7.0 ± 0.1	7.0 ± 0.1	6.9 ± 0.4	6.6 ± 0.2
*Σ*SFA^1^	28.7 ± 0.5	28.4 ± 0.3	28.4 ± 0.3	29.2 ± 0.0	29.2 ± 0.2	27.5 ± 0.3	28.4 ± 0.3	28.8 ± 0.5	28.0 ± 0.4	28.8 ± 0.1	29.0 ± 0.4	27.7 ± 0.4
C16:1*n*−7	3.8 ± 0.3	2.9 ± 0.3	2.8 ± 0.2	3.4 ± 0.6	3.6 ± 0.2	3.8 ± 0.3	3.5 ± 0.3	2.5 ± 0.1	3.4 ± 0.1	3.3 ± 0.2	3.4 ± 0.7	3.9 ± 0.4
C18:1*n*−9	24.5 ± 0.6	23.3 ± 0.7	22.4 ± 0.7	25.4 ± 0.7	24.4 ± 1.3	23.9 ± 0.4	23.8 ± 0.6	23.0 ± 0.6	23.4 ± 0.6	24.1 ± 2.6	26.2 ± 0.7	25.1 ± 1.1
*Σ*MUFA^2^	31.8 ± 0.9	29.3 ± 1.0	28.0 ± 1.1	31.7 ± 1.1	30.9 ± 1.7	30.7 ± 0.7	30.2 ± 0.7	28.4 ± 0.8	29.8 ± 0.7	30.4 ± 3.1	32.6 ± 1.2	31.8 ± 1.6
C18:2*n*−6	19.7 ± 0.9^bc^	21.0 ± 0.5^ab^	22.6 ± 0.0^a^	18.6 ± 0.6^bc^	18.2 ± 0.3^c^	21.0 ± 0.7^ab^	20.1 ± 0.5^abc^	19.4 ± 0.3^bc^	21.0 ± 0.3^ab^	19.0 ± 1.8^bc^	19.5 ± 0.1^bc^	20.8 ± 1.0^abc^
*Σn*6 FA^3^	21.8 ± 1.0	22.7 ± 0.7	24.2 ± 0.2	20.4 ± 0.4	20.1 ± 0.2	23.0 ± 0.8	22.3 ± 0.5	21.0 ± 0.2	22.8 ± 0.2	20.8 ± 2.1	21.5 ± 0.3	23.1 ± 1.0
C18:3*n*−3	2.4 ± 0.0	2.2 ± 0.1	2.3 ± 0.0	2.0 ± 0.0	1.8 ± 0.0	2.3 ± 0.1	2.2 ± 0.0	1.9 ± 0.0	2.2 ± 0.0	1.9 ± 0.3	2.1 ± 0.0	2.3 ± 0.1
C20:5*n*−3	3.6 ± 0.1	3.4 ± 0.1	3.1 ± 0.0	3.4 ± 0.1	3.5 ± 0.0	3.5 ± 0.2	3.4 ± 0.2	3.6 ± 0.1	3.3 ± 0.1	3.2 ± 0.3	3.2 ± 0.1	3.5 ± 0.2
C22:6*n*−3	11.4 ± 0.9	13.8 ± 0.9	13.7 ± 0.9	12.9 ± 0.8	14.1 ± 1.9	12.6 ± 1.1	13.1 ± 1.1	16.0 ± 1.2	13.6 ± 1.2	14.5 ± 5.2	11.3 ± 1.5	11.2 ± 1.9
*Σn*3 FA^4^	17.5 ± 1.0	19.5 ± 0.9	19.2 ± 0.9	18.4 ± 0.7	19.6 ± 1.7	18.6 ± 1.1	18.9 ± 1.2	21.7 ± 1.1	19.2 ± 1.2	19.9 ± 5.2	16.7 ± 1.5	17.2 ± 1.8
HUFA^5^	15.3 ± 1.0	17.4 ± 1.0	17.1 ± 0.9	16.4 ± 0.6	17.9 ± 1.8	16.4 ± 1.1	16.9 ± 1.3	19.7 ± 1.2	17.1 ± 1.2	18.1 ± 5.5	14.9 ± 1.6	15.1 ± 1.9
*Σn*3/*Σn*6	0.8 ± 0.0	0.8 ± 0.0	0.7 ± 0.0	0.9 ± 0.0	0.9 ± 0.0	0.8 ± 0.0	0.8 ± 0.0	1.0 ± 0.0	0.8 ± 0.0	1.0 ± 0.4	0.7 ± 0.0	0.7 ± 0.1

*Note*: Values are mean of triplicate groups and presented as mean ± SE. ^a,b,c^Different superscripts in the same row indicate significant differences between the means by ANOVA at *P* < 0.05. The lack of superscripts in the same row indicates no significant difference between the means by ANOVA at *P* > 0.05. ^1^Saturated fatty acids; ^2^monounsaturated fatty acids; ^3^*n*−6 polyunsaturated fatty acids; ^4^*n*−3 polyunsaturated fatty acids; ^5^highly unsaturated fatty acids. The fatty acids C12 : 0, C13 : 0, C14 : 1*n*−5, C15:0, C16:1*n*−9, C17:0, C17:1, C18:1*n*−7, C18:3*n*−6; C20:0; C20:1*n*−9; C20:2*n*−6, C20:3*n*−6, C20:3*n*−3, C20:4*n*−6, C20:4*n*−3, C22:0, C22:1*n*−9, C22:2*n*−6, C22:5*n*−3, and C24:0 in percentage ≤ 1%, were also detected and used to calculate the fatty acid groups.CON, 60% FM; SPC, 30% FM with SPC; CG, 30% FM with CG; MM, 30% FM with MM; CM, 30% FM with CM; SCG, 30% FM with SPC and GC; SMM, 30% FM with SPC and MM; SCM, 30% FM with SPC and CM; CMM, 30% FM with CG and MM; CCM, 30% FM with CG and CM; MCM, 30% FM with MM and CM; MIX, 30% FM with SPC, GC, MM, and CM.

**Table 10:** Fillet essential amino acid composition of red seabream fed the experimental diets for 12 weeks (% of protein).

	Experimental diets											
	CON	SPC	CG	MM	CM	SCG	SMM	SCM	CMM	CCM	MCM	MIX
Arg	6.2 ± 0.1	6.3 ± 0.0	6.2 ± 0.1	6.3 ± 0.1	6.3 ± 0.0	6.3 ± 0.0	6.3 ± 0.0	6.4 ± 0.0	6.3 ± 0.1	6.4 ± 0.0	6.3 ± 0.0	6.4 ± 0.0
His	2.8 ± 0.2	2.7 ± 0.2	2.6 ± 0.1	2.6 ± 0.1	2.6 ± 0.0	2.6 ± 0.0	2.6 ± 0.0	2.6 ± 0.1	2.7 ± 0.1	2.6 ± 0.0	2.6 ± 0.0	2.7 ± 0.0
Ile	4.3 ± 0.1	4.3 ± 0.2	4.3 ± 0.1	4.3 ± 0.1	4.5 ± 0.1	4.5 ± 0.0	4.6 ± 0.1	4.5 ± 0.1	4.4 ± 0.1	4.5 ± 0.0	4.4 ± 0.0	4.4 ± 0.1
Leu	8.8 ± 0.2	8.7 ± 0.3	9.0 ± 0.1	8.9 ± 0.1	8.9 ± 0.0	9.0 ± 0.0	8.9 ± 0.0	8.9 ± 0.1	8.8 ± 0.2	8.9 ± 0.1	9.2 ± 0.1	9.2 ± 0.2
Lys	9.5 ± 0.3	9.4 ± 0.4	9.6 ± 0.2	9.7 ± 0.1	9.8 ± 0.0	9.9 ± 0.0	9.9 ± 0.1	9.8 ± 0.0	9.7 ± 0.1	9.8 ± 0.0	9.8 ± 0.0	9.7 ± 0.1
Met + Cys	4.2 ± 0.2	4.1 ± 0.1	4.1 ± 0.0	4.1 ± 0.0	4.0 ± 0.0	3.9 ± 0.1	4.1 ± 0.1	3.9 ± 0.0	4.1 ± 0.2	4.0 ± 0.1	4.0 ± 0.1	3.9 ± 0.0
Phe	4.2 ± 0.0	4.1 ± 0.0	4.1 ± 0.1	4.1 ± 0.1	4.1 ± 0.0	4.1 ± 0.0	4.1 ± 0.0	4.1 ± 0.0	4.0 ± 0.1	4.1 ± 0.0	4.2 ± 0.0	4.2 ± 0.0
Thr	4.7 ± 0.1	4.7 ± 0.1	4.7 ± 0.1	4.7 ± 0.1	4.6 ± 0.0	4.6 ± 0.0	4.6 ± 0.0	4.6 ± 0.0	4.7 ± 0.1	4.6 ± 0.0	4.6 ± 0.0	4.7 ± 0.1
Val	4.8 ± 0.2	4.8 ± 0.2	4.8 ± 0.1	4.9 ± 0.1	5.1 ± 0.0	5.0 ± 0.0	5.1 ± 0.1	5.0 ± 0.1	5.0 ± 0.0	5.1 ± 0.1	5.0 ± 0.1	5.0 ± 0.1

*Note*: Values are mean of triplicate groups and presented as mean ± SE. CON, 60% FM; SPC, 30% FM with SPC; CG, 30% FM with CG; MM, 30% FM with MM; CM, 30% FM with CM; SCG, 30% FM with SPC and GC; SMM, 30% FM with SPC and MM; SCM, 30% FM with SPC and CM; CMM, 30% FM with CG and MM; CCM, 30% FM with CG and CM; MCM, 30% FM with MM and CM; MIX, 30% FM with SPC, GC, MM, and CM.

**Table 11:** Biochemiical parameters of red seabream fed the experimental diets for 12 weeks.

	Experimental diets											
	CON	SPC	CG	MM	CM	SCG	SMM	SCM	CMM	CCM	MCM	MIX
GOT^1^	10.0 ± 3.2	12.3 ± 4.1	11.0 ± 0.0	11.3 ± 3.4	9.0 ± 1.2	11.7 ± 1.5	13.0 ± 3.1	9.3 ± 3.0	11.0 ± 2.3	7.3 ± 1.9	11.3 ± 0.3	8.7 ± 1.2
GPT^2^	4.00 ± 0.0	4.33 ± 0.3	4.00 ± 0.0	4.00 ± 0.0	3.67 ± 0.3	3.67 ± 0.3	4.00 ± 0.58	4.33 ± 0.3	4.00 ± 0.0	4.00 ± 0.0	4.00 ± 0.0	4.00 ± 0.0
TBIL^3^	0.10 ± 0.0	0.17 ± 0.0	0.17 ± 0.0	0.17 ± 0.0	0.13 ± 0.0	0.13 ± 0.0	0.13 ± 0.0	0.13 ± 0.0	0.17 ± 0.0	0.13 ± 0.0	0.13 ± 0.0	0.13 ± 0.0
TP^4^	2.60 ± 0.3	3.37 ± 0.1	3.20 ± 0.1	3.10 ± 0.1	3.10 ± 0.1	3.20 ± 0.1	3.17 ± 0.3	2.27 ± 0.8	3.07 ± 0.5	2.70 ± 0.4	3.17 ± 0.2	3.27 ± 0.1
ALB^5^	0.53 ± 0.1	0.63 ± 0.1	0.63 ± 0.0	0.60 ± 0.1	0.60 ± 0.1	0.63 ± 0.0	0.63 ± 0.1	0.43 ± 0.2	0.63 ± 0.1	0.50 ± 0.1	0.67 ± 0.0	0.60 ± 0.0
GLU^6^	59.0 ± 2.5	73.3 ± 20.9	45.7 ± 8.7	45.7 ± 6.7	46.3 ± 3.8	47.3 ± 4.9	53.7 ± 2.2	74.0 ± 26.6	51.7 ± 2.3	46.3 ± 5.4	50.3 ± 2.7	54.0 ± 3.5
NH_3_^7^	263.0 ± 10	372.0 ± 68	421.0 ± 79	301.7 ± 38	310.3 ± 14	365.3 ± 68	297.0 ± 23	243.3 ± 18	334.3 ± 38	341.7 ± 16	289.7 ± 22	395.0 ± 79
AMYL^8^	7.33 ± 1.9	9.33 ± 0.9	8.33 ± 2.3	6.33 ± 0.9	6.67 ± 1.20	7.00 ± 1.2	9.00 ± 1.5	7.67 ± 1.9	7.00 ± 1.1	6.33 ± 1.9	10.0 ± 1.0	7.33 ± 1.7
TCHO^9^	159.3 ± 54	141.0 ± 15	105.7 ± 12	117.3 ± 17	155.0 ± 10	128.7 ± 5.2	145.3 ± 17	101.3 ± 32	124.7 ± 18	106.0 ± 9.9	153.7 ± 8.4	174.3 ± 16

*Note*: Values are mean of triplicate groups and presented as mean ± SE. ^1^Glutamic oxaloacetic transaminase (U/L); ^2^glutamic pyruvic transaminase (U/L); ^3^total bilirubin (mg/dL); ^4^total protein (g/dL); ^5^albumin (mg/dL); ^6^glucose (mg/dL); ^7^ammonia (µg/dL); ^8^amylase (U/L); ^9^total cholesterol (mg/dL). CON, 60% FM; SPC, 30% FM with SPC; CG, 30% FM with CG; MM, 30% FM with MM; CM, 30% FM with CM; SCG, 30% FM with SPC and GC; SMM, 30% FM with SPC and MM; SCM, 30% FM with SPC and CM; CMM, 30% FM with CG and MM; CCM, 30% FM with CG and CM; MCM, 30% FM with MM and CM; MIX, 30% FM with SPC, GC, MM, and CM.

**Table 12:** Selected nonspecific immune and antioxidant enzyme activities of red seabream fed the experimental diets for 12 weeks.

	Experimental diets											
	CON	SPC	CG	MM	CM	SCG	SMM	SCM	CMM	CCM	MCM	MIX
SOD^1^	78.5 ± 0.5	83.7 ± 3.3	83.8 ± 3.4	85.3 ± 0.6	84.1 ± 4.0	83.3 ± 2.4	80.9 ± 2.4	85.8 ± 4.4	82.9 ± 1.3	80.9 ± 5.6	92.4 ± 6.1	80.0 ± 4.1
Lysozyme^2^	283 ± 22^b^	217 ± 61^b^	160 ± 47^b^	300 ± 40^b^	343 ± 59^b^	280 ± 11^b^	270 ± 36^b^	137 ± 19^b^	150 ± 59^b^	233 ± 47^b^	950 ± 298^a^	867 ± 328^a^

*Note*: Values are mean of triplicate groups and presented as mean ± SE. ^a,b^Different superscripts in the same row indicate significant differences between the means by ANOVA at *P* < 0.05. The lack of superscripts in the same row indicates no significant difference between the means by ANOVA at *P* > 0.05. ^1^Superoxide dismutase (% inhibition); ^2^lysozyme (U/mL). CON, 60% FM; SPC, 30% FM with SPC; CG, 30% FM with CG; MM, 30% FM with MM; CM, 30% FM with CM; SCG, 30% FM with SPC and GC; SMM, 30% FM with SPC and MM; SCM, 30% FM with SPC and CM; CMM, 30% FM with CG and MM; CCM, 30% FM with CG and CM; MCM, 30% FM with MM and CM; MIX, 30% FM with SPC, GC, MM, and CM.

## Data Availability

The data that support the findings of this study are available from the corresponding author upon reasonable request.
